# Supplementary material for: Uncovering miRNA-mRNA Regulatory Modules in Developing Xylem of Pinus massoniana via Small RNA and Degradome Sequencing
Source: Int J Mol Sci. 2021 Sep 21;22(18):10154. doi: 10.3390/ijms221810154 (PMC8472836; doi:10.3390/ijms221810154)
Supplement: Supplementary file 1 [file ijms-22-10154-s001.zip › ijms-1365557-supplementary/Additional file S2.zip!/Additional file 2/pma-miR1314_Cluster-18410.30775_161_TPlot.pdf]

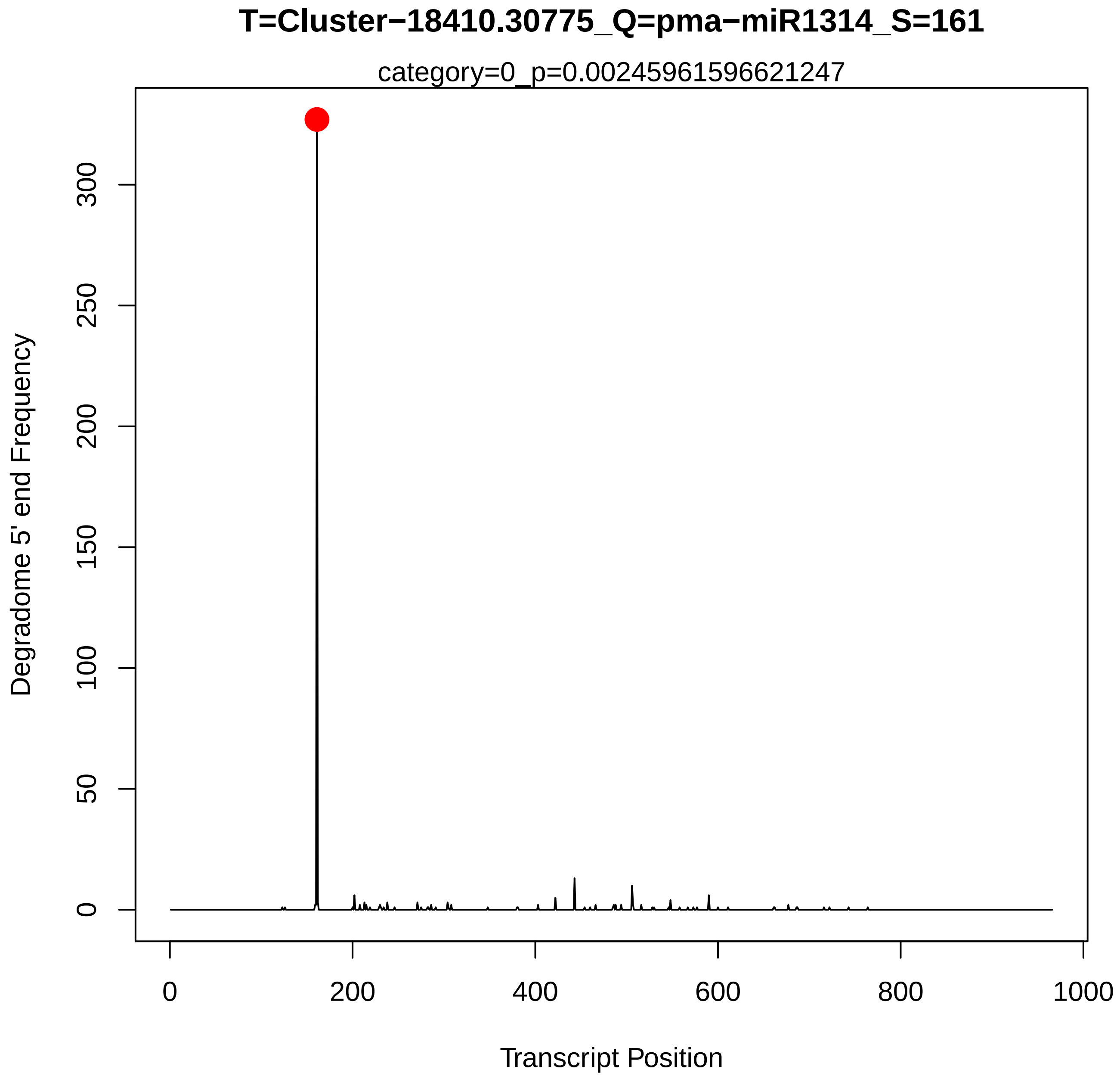
T=Cluster−18410.30775_Q=pma−miR1314_S=161
category=0_p=0.00245961596621247
Degradome 5' end Frequency
0
50
100
150
200
250
300
0
200
400
600
800
1000
Transcript Position
